# Emergency contraception subsidy in Canada: a comparative policy analysis

**DOI:** 10.1186/s12913-022-08416-1

**Published:** 2022-09-01

**Authors:** Sabrina C. Lee, Wendy V. Norman

**Affiliations:** 1 Faculty of Epidemiology and Population Health, London School of Hygiene & Tropical Medicine, London, UK; 2 Department of Obstetrics, Gynecology and Reproductive Sciences, University of Manitoba, WN5002-665 William Ave, Winnipeg, MB R3E 0L8 Canada; 3 Faculty of Public Health & Policy, London School of Hygiene & Tropical, London, UK; 4 Department of Family Practice, University of British Columbia, 320-5950 University Boulevard, Vancouver, BC V6T 1Z3 Canada

**Keywords:** Ulipristal acetate, Emergency contraception, Universal subsidy, Canada, Health policy, Evidence-based policy

## Abstract

**Background:**

In Canada, cost prohibits access to emergency contraception (EC) which may assist to prevent unintended pregnancy. The drug, ulipristal acetate (UPA-EC), is more clinically effective and cost-effective than the prior standard levonorgestrel (LNG-EC). We analyzed provincial EC subsidization policies and examined underlying decision-making processes.

**Methods:**

We undertook documentary analysis of provincial EC subsidization policies in publicly available drug formularies. We conducted semi-structured interviews with key informants to explore the processes underlying current policies.

**Results:**

Quebec is the only province to subsidize UPA-EC, whilst all ten provinces subsidize LNG-EC. As such, provincial EC subsidization policies do not align with the latest UPA-EC evidence. Interviews revealed that evidence was valued in the policymaking process and formulary decisions were made through interdisciplinary consensus.

**Conclusions:**

We identify a gap between EC subsidization policies and the latest evidence. Institutional structures affect policies reflecting evolving evidence. Increasing interdisciplinary mechanisms may encourage evidence-based policies.

## Background

Unintended pregnancy can be life-altering. Medical advances offer several methods to assist people to prevent untimely pregnancy, which can lead to increased autonomy for those at risk.

Among family planning methods, emergency contraception (EC) may be taken to attempt to prevent pregnancy from occurring after unprotected vaginal intercourse, contraception failure, or sexual assault. The effectiveness of this class of medication decreases within days of the event leading to progressively higher risk of unintended pregnancy [1]. Due to their narrow window of use, access to these drugs influences their effectiveness.

Currently, levonorgestrel (LNG-EC) is the most used and well-known oral method of emergency contraception [2]. However, another oral agent, ulipristal acetate (UPA-EC), has been found to be more clinically effective than LNG-EC, particularly in women with higher body weight, and to have similar side effects [3]. Other emergency contraceptives include the copper intrauterine device (Cu-IUD) (most effective to prevent pregnancy), the Yuzpe method (combination of oral contraceptive pills) and mifepristone (unavailable for this indication in Canada) [4, 5].

### Canadian healthcare system

In Canada, there is universal access to “medically necessary” healthcare; however, this excludes services such as medications, physiotherapy, dental health, and eye care [6, 7]. Health care is mandated by the Federal government, but health care and pharmaceutical services are delivered by each provincial government independently [8]. As a result, access to pharmaceutical therapies can be variable and inequitable. To address this issue, provincial health insurance plans offer subsidies to some of those without private drug insurance plans. However, important gaps in coverage exist, with a reported 4.1 million people not enrolled in eligible programs due to lack of awareness, lack of need, or out-of-pocket premiums [9]. As such, individuals in the second quintile of household income, such as low wage single parents or people with precarious and occasional employment, paid disproportionately more out-of-pocket on drugs due to their borderline income level. Populations at the margin of income assistance may be particularly affected by an unintended pregnancy.

Drugs subsidized by provincial insurance plans are listed in each province’s publicly available formularies. To be listed on a formulary first requires approval by Health Canada for distribution and sale in the country [10]. Provincial governments then refer to reports like the Common Drug Review (CDR) which independently compiles evidence on comparators, costs and outcomes and makes subsidization recommendations to assist their decision making toward providing a provincial subsidy [11]. This process aligns with the health technology assessment model which encourages multifaceted decision-making in healthcare delivery [12]. The CDR was established to standardize the drug approval process across the country, but inquiries have shown variable levels of agreements between CDR recommendations and provincial formulary listings [13–16]. Ultimately, this system results in drug coverage varying depending on provincial residence, employment status, marital status, and income, which leads to uneven access to contraception across the country.

### Family planning and emergency contraceptives in Canada

Approximately one third of Canadian women have an induced abortion in their reproductive lifetime [17]. According to a 2015/2016 Canadian Community Health Survey, 24.9% of women who were not pregnant and did not wish to become pregnant reported not using any form of contraception the last time they engaged in sexual intercourse [18]. Black et al. [19] estimated there were 180,733 unintended pregnancies in 2015, directly costing the healthcare system CAD$320 million, of which 69% was attributed to imperfect use or failure of contraceptive methods. Some of these costs could be avoided by increased use of EC methods

Contextually, Canada offers fewer contraceptive options with longer approval times for new technologies, compared to the United States, the United Kingdom, and the European Union [20]. HRA Pharma introduced UPA-EC (brand name: ella®) for emergency contraception to the Canadian market in 2015 [21]. This introduction came shortly after Health Canada released an advisory warning of the reduced or absent effectiveness of LNG-EC in women weighing over 75 kg or 80 kg, respectively [22]. The proportion of Canadian women who are considered overweight or obese was estimated at 56.7% in 2018 [23]. Therefore, half of Canadian women may be considered by their prescriber or dispenser to be ineligible for LNG-EC, the gold-standard oral EC since 2000, due to their weight [24].

The Canadian Agency for Drugs and Technologies in Health (CADTH) has released two reports finding UPA-EC to be more clinically effective and cost-effective than LNG-EC [25, 26]. Despite this evidence, barriers to access the drug persist. The cost of contraceptives has been identified as the most important barrier to family planning in Canada [27]. For eligible key populations, publicly funded provincial drug plans subsidize the cost of medications listed on their respective formularies [28]. Considering the established need for improved access to effective family planning methods, particularly in women weighing more than 75 kg, this project aims to examine provincial EC subsidization policies and explore their decision-making processes. We aimed to determine the current policy decisions, and the processes surrounding UPA-EC subsidy, as this drug is the most effective oral EC method for this key population.

## Methods

### Documentary analysis

#### Document selection

We acquired documents on EC subsidization policies in each province using publicly available drug formularies. We carefully searched provincial government websites for details of 1) the key populations covered by these insurance plans, 2) the emergency contraceptives covered and 3) the process to obtain coverage. We limited this search to information on LNG-EC, UPA-EC, and the copper intrauterine device (Cu-IUD).

We undertook a broader Google search for public-domain websites presenting information on how to access EC in each province. We used search terms such as “emergency contraception [name of province]” and “how much does [EC method] cost in [name of province]”. We designed this strategy to represent real-life circumstances of reproductive age Canadians requiring EC searching the internet for information on costs and availability in their province. We performed the searches from June to July 2020 in Winnipeg, Manitoba.

#### Data collection

We extracted details of each province’s EC subsidization policies and organized these data into a Microsoft Excel spreadsheet. The choice of which data to extract was initially guided by deductive reasoning informed by the hypothesis and background reading. Examples of pre-established data points included which emergency contraceptives were covered, how much they cost, under which plan they were covered, which populations were eligible for these plans and what process was involved in choosing which drugs to subsidize. As findings emerged from the documentary search and semi-structured interviews, an iterative process took place in collecting additional data points.

#### Data analysis

We compared pertinent elements of each province’s EC subsidization policies to each other and to the established EC literature. We tabulated descriptive data to facilitate comparison. We analyzed the provincial subsidization policies using the policy triangle theoretical framework [29]. Conceptualizing health policy through actors, content, process, and context informed our findings.

### Key informant semi-structured interviews

#### Participant selection and recruitment

We selected a sample of three provinces (Quebec, Ontario, British Colombia) based on their EC subsidization policies to obtain insights into underlying decision-making processes. We recruited a purposive sample of provincial government, national organization, and academic leaders in the field of family planning policymaking. We invited participants through email correspondence.

Following a positive response, and once voluntary written consent was documented, we scheduled interviews at the participants’ earliest conveniences.

#### Data collection

We conducted interviews in English and French over Zoom (version 5.0.2) videoconference in July 2020. Fluently bilingual, the interviewer (SL) has clinical experience as an Obstetrics and Gynecology resident physician training in Canada. After answering questions and obtaining explicit verbal consent via video-linked conversation, SL audio-recorded interviews to assist with analysis. SL asked open-ended questions on provincial EC subsidization policies, awareness of the evidence on UPA-EC and knowledge of the actors, processes, and context regarding these enacted policies. Participants could retract statements or discontinue the interview at any point during the discussion without any consequence to them. Once the interviews concluded, SL stopped audio-recording and stored recordings on an encrypted password-protected laptop.

#### Data analysis

Once the interviews were completed, SL transcribed the audio-recordings and analyzed the qualitative data in an iterative process spanning the entirety of the project’s timeline. SL performed thematic analysis using an inductive “working up” approach which allowed the collected qualitative data to organically generate ideas [30]. This qualitative approach seemed most appropriate as these interviews sought to further understand the processes behind these subsidization decisions. Once transcribed, SL listened to the audio-recordings repeatedly to detect important moments in the conversations. SL identified codes manually using Microsoft Word, then used NVivo 12 (version 12.6.0) to analyze preliminary results. SL labelled categories as they arose from the established codes. Finally, SL developed themes from these categories and reviewed them against the data once again.

### Ethics

Data for the documentary analysis were public domain or academically accessible content. We obtained voluntary and informed written and verbal consent prior to scheduling and audio-recording the semi-structured interviews, respectively. We conducted interviews on an encrypted videoconference platform (Zoom) and anonymized data from the interviews to preserve participant confidentiality. We encrypted and stored all documents produced on a password-protected laptop.

### Theoretical framework

We designed this policy analysis under the interpretivist premise that policies develop through the interaction between a defined set of important factors [31]. The policy triangle framework introduced by Walt & Gilson [29] emphasizes an approach that considers the roles of content, process, actors, and context in the analysis of a specific policy. This framework informed the research question, data prioritization, and conceptual approach to analyzing and interpreting the findings in this study.

We conducted this study from an anti-oppressive perspective [32, 33]. Recognition of family planning’s roots in eugenics, colonialism, and white supremacy is crucial to fully implementing a rights-based approach to contraception and abortion care [34].

## Results

Results from the documentary search and the semi-structured interviews informed this policy analysis. We found complete policy positions for all ten Canadian provinces and did not include the territories in our search (Table 1).

**Table 1:** Emergency contraception subsidization policies in Canadian provinces

Province	Emergency contraceptive subsidized			Key populations eligible for EC subsidization [35]	Prescription status [36]			Cost without subsidy (CAD$)		
	LNG-EC	UPA-EC	Cu-IUD		LNG-EC	UPA-EC	Cu-IUD	LNG-EC	UPA-EC	Cu-IUD
BritishColumbia [37]				Low income/receiving provincial income assistance, children with severe disabilities, First Nations individuals* (only plan to cover Cu-IUD)	OTC	P	HCP	15–40 [38, 39]	27–45 [38, 39]	75–150 [38, 39]
Alberta [40]				Low income adults, age 65 and older, children under child services or in low income families	OTC	P	HCP	21.59–33.49 [41]	-	76.50 [41]
Saskatchewan [42]				Any resident with Saskatchewan Health Coverage	BTC	P	HCP	21.92 [43]	42.35 [43]	70–140 [43]
Manitoba [44]				Any resident not eligible for other provincial or federal plan (income-based deductible)	OTC	P	HCP	20–40 [45]	-	-
Ontario [46]				Youth (under age 25), age 65 and older, people in long term care homes, receiving home care services or spending large proportion of income on drugs	OTC	P	HCP	25–42.25 [47, 48]	-	40–50 [47, 48]
Quebec [49]				Any resident who does not have access to private insurance through their employer (mandatory)	BTC/P	P	HCP	26 [50]	20–90 [51]	35 [52]
Nova Scotia [53]				Income-based program for residents without other coverage, long term care residents under 65 years, low income residents receiving income assistance	OTC	P	HCP	-	-	80 [54]
New Brunswick [55]				Any resident with New Brunswick Medicare card and without private insurance	OTC	P	HCP	28.69 [56]	-	85 [57]
Prince Edward Island [58]				Low income families with at least one child, people who have exceeded a predetermined out-of-pocket drug expenses limit, people receiving social assistance, aged 65 and older who do not have private insurance	OTC	P	HCP	-	-	-
Newfoundland & Labrador [59]				Low income families, 65 and older, anyone who spends an increased percentage of their income on drug costs (different percentage depending on income)	OTC	P	HCP	-	-	-

*OTC* Over-the-counter

*BTC* Behind-the-counter

*P* Prescription only

*HCP* Insertion by healthcare professional

*LNG-EC* Levonorgestrel emergency contraception

*UPA-EC* Ulipristal acetate emergency contraception

*Cu-IUD* Copper intrauterine device

= subsidized,  = subsidized with limitations,  = not subsidized

We conducted four interviews lasting 5–24 min with key informants from the three largest provinces. These key informants occupied several roles within the policymaking process (public health and clinical researcher, leadership within professional associations, public health agency management, clinical practice). Data saturation was not reached as this purposive sample continuously contributed novel data [60].

### Content

As the only province to subsidize UPA-EC, Quebec and their Free Emergency Oral Contraception Services Program aligned most with the latest evidence on EC [49, 51]. British Columbia was the only province to offer some coverage for the Cu-IUD [37]. Otherwise, no other province demonstrated EC subsidisation policies consistent with the latest EC evidence.

All provinces subsidized between CAD$8.60–17.53 of the LNG-EC cost, depending on the brand dispensed. Manitoba, Saskatchewan, Quebec, and New Brunswick offered provincial drug subsidization to broad groups of residents as opposed to specific populations [35]. Provinces with precise eligibility criteria targeted individuals who had low incomes, high drug expenditures or special needs. Ontario was distinct by broadly covering youth aged under 25 years [61]. Post-menopausal women above 65 years were specifically listed as a key population eligible for EC coverage in Alberta, Ontario, Nova Scotia, Prince Edward Island and Newfoundland & Labrador.

In terms of acquiring EC, Quebec and Saskatchewan stood apart by making all EC methods available by prescription only, with pharmacists and nurses being able to prescribe them. Pharmacists are permitted to provide EC in Alberta, Saskatchewan, Quebec, New Brunswick, Nova Scotia, Prince Edward Island and Newfoundland & Labrador [62, 63]. Provinces without pharmacist-prescribed EC (British Colombia, Manitoba, Ontario) offered LNG-EC over-the-counter but required prescription for UPA-EC. Nurse practitioners, characterized by their advanced clinical scope of practice, can prescribe EC in all provinces [64]. Since 2018, British Columbia, Saskatchewan, Alberta and Manitoba have created various nurse prescriber designations allowed to prescribe contraception with additional specialized training [65–68]. Contrastingly, Quebec has enabled registered nurses to prescribe all contraceptives using collaborative agreements since 2007 [69, 70].

When paying for EC out-of-pocket, the Women’s College Hospital website “What’s next for me” quoted national prices of UPA-EC, LNG-EC and Cu-IUD at CAD$40–50, CAD$30–40 and CAD$70–80, respectively [71]*.* Online information on respective EC drug pricing varied between provinces. We found details on costs most often on university clinic or sexual health clinic websites.

### Actors

Actors can be divided as playing a central role in decision-making or peripherally influencing the decision to subsidize an emergency contraceptive.

Provincial governments and their public health agencies set the formulary by compiling and evaluating the evidence and recommendations. At the federal level, Health Canada and CADTH approve the use and recommend the subsidization of each submitted medication, respectively. Drug manufacturers instigate the entire approval and subsidization processes. Overwhelmed with the number of requests, CADTH started requiring applications fees for the Common Drug Review (CDR) in September 2014 [72].

The manufacturer of UPA-EC did not commission a CDR which halted the decision process for subsidy in most provinces. Marketed in 2015, the CDR application fee for ella® would have cost over CAD$72,000 [72]. At the time, HRA Pharma was a Paris-based pharmaceutical company focusing on reproductive health and rare disease drugs in global markets [73, 74]. Currently leading EC provision in Europe, their only presence in Canada to date is through selling the UPA-EC drug marketed as ella® [75, 76]. Other than ella®, their history within the Canadian market is limited to the LNC-EC drug named Norvelo®, with an accepted 1.5 mg formulation that is not yet marketed and a 0.75 mg formulation that was cancelled post-market in 2017 [77, 78].

Healthcare professionals interact with the decision-making process by acting within the structure of provincial committees or engaging through advocacy and knowledge production. Their professional associations may influence decision-making by publicly supporting positions through statements to their members and communications to provincial governments. For example, the Canadian Federation of Nurses Unions published an extensive report advocating for the universal publicly-funded coverage of prescription drugs in the country [79]. Specific to contraception, the Canadian Pediatrics Society and Society of Obstetricians and Gynecologists of Canada have both encouraged the free provision of all contraceptives [4, 80]. Similarly, non-governmental organizations such as Action Canada for Sexual Health & Rights or local grassroots movements like AccessBC have been involved in supporting national policy change relative to contraception coverage through advocacy and pressure tactics [81–83].

### Process

**Fig. 1:**
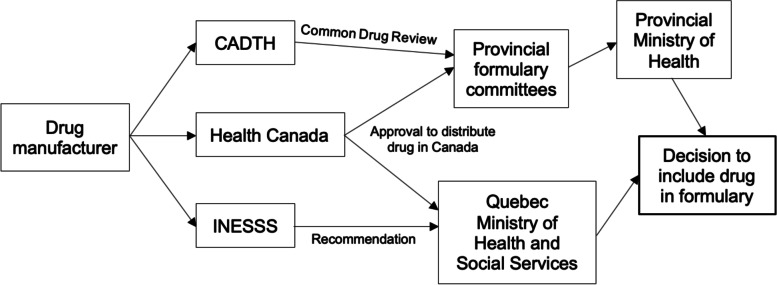
Drug subsidization process in Canada

The subsidization process was similar across most provinces (Fig. 1). Once approved by Health Canada, multidisciplinary advisory committees (such as the Committee to Evaluate Drugs in Ontario) convene to decide on the inclusion of new pharmaceuticals in their province’s formulary by reviewing the scientific evidence and CADTH recommendations [84]. These specialized formulary committees then make a recommendation to their provincial Ministry of Health to include the drug to their respective formulary. Larger provinces may conduct further research to tailor their conclusions to their regional context. Quebec was demarcated by resorting to their own health technology assessment agency (INESSS), which also served as the actor providing formulary recommendations directly to the Quebec Ministry of Health and Social Services.

Specific to UPA-EC, Quebec’s process to attain this policy decision provides valuable insight. Prior to Canada’s shift towards over-the-counter LNG-EC, Quebec’s public health professionals facilitated provincial law reform to enable pharmacists to prescribe EC. Gaining momentum, they obtained permissions for nurses and pharmacists to prescribe all EC methods and regular hormonal contraceptives. When UPA-EC became available, nurses noticed a discrepancy between provincial subsidization policies and the *Institut national de santé publique du Québec* (INSPQ) clinical guidelines, which often favoured UPA-EC due to its superior effectiveness particularly in people with higher weights. Eventually, nursing professionals brought forward a complaint to the *Institut national d’excellence en santé et services sociaux* (equivalent to CADTH in Quebec) which triggered the process to subsidize UPA-EC.

### Context

These EC subsidization policies highlighted the importance of context and temporality in policymaking. Quebec nurses being involved in EC prescription led to the eventual subsidization of UPA-EC. Furthermore, Quebec’s policy enabling pharmacists to directly prescribe EC contributed to their decision to maintain the prescription status of EC.

Normative context influences these policies. While searching for readily available information on EC cost and access, information regarding family planning became notably scarcer in less affluent, more socially conservative provinces like New Brunswick and Prince Edward Island. These norms are exemplified in their abortion policies with out-of-pocket expenses ranging CAD$700–850 in New Brunswick for abortion care and abortion becoming available for the first time in Prince Edward Island in 2017 [57, 85].

### Additional themes

#### Scientific evidence important in making formulary recommendations

High-quality evidence held a prominent role within the health technology assessment used to guide the decision-making process. When determining whether to include drugs on formularies, dedicated committees were tasked with assessing scientific literature and recommendations from respected bodies. When asked about the process to change policy, bringing forward new compelling evidence was often described as the instigating step. Current evidence *“would make a good case to consider adding this [UPA-EC] to the formulary”* (key informant #3, British Columbia).

#### Interdisciplinary networks central to decision-making process

Relevant decision-making committees were consistently composed of actors from various professions and perspectives. Consensus and collaboration were recurrent requirements within the decision-making process. Additionally, when asked about determining factors in deciding on drug subsidization, there was indication that policies in other jurisdictions were considered:

*“I think they would also like to know what are other jurisdictions doing. So, what’s happening, is this drug covered in other provinces and territories? Is it covered in other similar health systems? They usually look to the UK, Australia, and New Zealand as kind of comparable places for a lot of our health systems planning. So, the jurisdictional scan would be important.”* (key informant #3, British Columbia)

#### Concern for barriers to accessing EC

Multiple barriers to accessing EC such as cost, lack of provider knowledge and abortion stigma were identified. One key informant commented on the limitations of provincial insurance plans in relieving the burden of cost:

*“I’m sure you can find out what percentage of women you know under the age of say 50 it actually covers and I would anticipate it would be actually very low… There hasn’t been a lot of cost coverage. Most of the time it has been out-of-pocket costs for the individual.”* (key informant #4, British Columbia)

Despite recognizing the cost of EC as potentially prohibitive, budgetary concerns and fiscal responsibility were prioritized when deciding whether to subsidize a drug.

## Discussion

### Formulary alignment with latest UPA-EC evidence

This project is the first to demonstrate that, while the evidence indicates UPA-EC prevents unintended pregnancy more effectively than LNG-EC, Canadian provincial policies do not subsidize the cost of UPA-EC, except for Quebec. Most provincial EC subsidization policies did not reflect the high-quality evidence finding UPA-EC to be more clinically and cost-effective than LNG-EC [86–94]. Our results are consistent with the Patented Medicine Prices Review Board report on public formulary alignment, which found that public plans aligned less with each other for drugs with only one manufacturer (UPA-EC) than with drugs marketed by two or more manufacturers (LNG-EC) [95]. Quebec listed the greatest number of selected single-source drugs (*n* = 210/262; 80%) with provinces averaging 176/262 (67%) single-source drugs on their formulary [95].

Institutional structures may have contributed to Quebec formulary policies promptly reflecting the evolving EC literature. Notably, prescribing practices and compulsory mixed public/private payer system have contributed to Quebec spending more on prescription drugs per capita than any other province [96]. Additionally, Quebec includes the greatest number of drugs on their formulary (*n* = 628) compared to all other provinces, with provinces averaging 528 formulary listings out of the 729 investigated drugs [95].

Most provincial EC subsidization policies aligned with available CDR recommendations, with Quebec as an outlier. As the only province not following CADTH, Quebec did not rely on the release of a CDR recommendation to consider subsidizing UPA-EC. The absence of a CDR on UPA-EC as an emergency contraceptive potentially led other provinces to be unaware of the evidence supporting the benefits of improved access to UPA-EC. Nonetheless, our findings suggest that, should a CDR be initiated, the process of making a recommendation to include UPA-EC on formulary would involve reviewing the latest evidence and reaching multidisciplinary expert consensus. Recent publication of two rapid review reports from CADTH may indicate ongoing mechanisms to initiate UPA-EC subsidization [25, 26].

Our findings were consistent with findings in Hulme et al. [27], which demonstrate that Canadians continue to face the barrier of cost in accessing effective EC methods, particularly women with higher body weights. When investigating UPA-EC uptake from 2015–2018 in British Colombia, Chan et al. [97] identified few dispensations compared to LNG-EC relating to systemic barriers such as cost, prescription status, and fewer pharmacies carrying UPA-EC in stock due to perceived low demand.

### Policymaking mechanisms

Through speaking with key informants, we discovered that, when manufacturers or knowledge brokers trigger the established processes, formulary decisions were made through a collaborative process, which sought consensus within multidisciplinary committees and considered the available evidence and budgetary concerns. Interdisciplinary mechanisms facilitated the enaction of political change, resulting in different contexts and policy contents, as demonstrated by Quebec and their innovative collaborative approach to contraceptive prescribing [98]. This apparent advantage to task-sharing can be contrasted to reported challenges with trained nurses having difficulty finding opportunities to practice their new prescribing skills [99, 100]. Nonetheless, Quebec and British Colombia have the lowest rates of unmet contraceptive need (21.2% and 21.4%, respectively) compared to Prince Edward Island reporting the highest rate (30.6%) of sexually active women not using contraception and not wishing to become pregnant [18]. With more provinces considering registered nurses for contraception prescription, the effect of interdisciplinary mechanisms on systemic awareness of the latest evidence may be increasingly seen [101–103].

Differences in plan design, provincial demographics, and eligibility profiles influence formulary inclusion across provinces [95]. Provincial variation in prescribing scope of practice for pharmacists and nurses may have influenced whether to provide EC as an over-the-counter, behind-the-counter, or prescription only drug. Considered an important employer in the province, the pharmaceutical industry in Quebec was accommodated in the policymaking process behind the mandatory private/public plan, with compromises such as longer patent protection and minimal cost control, which led to steadily rising expenditures per capita on prescription drugs [104, 105]. The relationship between Quebec government and pharmaceutical industry may contribute to the broader and more costly coverage of prescription drugs [105]. As such, the health technology assessment mechanisms to include UPA-EC to the formulary may have been facilitated within this economical context.

### Implications of the Common Drug Review process

This project outlines a limitation of the centralized CDR process as the steps to initiate this report after a drug has been formally introduced remain unclear. If manufacturers do not instigate the CDR at product launch, an unspecified knowledge broker must then raise the value of possible subsidization to the attention of governments [106]. According to the CADTH Fee Schedule document, “Application fees will not apply to any submission, resubmission, or request for advice filed by the public drug programs or tumour groups.” [107] This fee exemption may support the role for governments to take the lead on triggering the CDR process should the evidence on a drug evolve. In the case of UPA-EC, the timing CADTH implementing application fees for the CDR process in 2014, as UPA-EC was marketed shortly after, in 2015, may have contributed to HRA Pharma bypassing this process when introducing their product to Canada [21, 72].

The CDR was created to streamline and simplify access to rigorous health technology assessments for all provinces; however, the problematization of the drug subsidization process in Canada may have led to the creation of another impediment [15, 108]. With an 81% average agreement between formulary listing and CDR recommendation [109], although the provinces are more unified (as shown by the associated 7% standard deviation in agreement), their policies may be unreflective of the latest evidence due to the institutionalism created by this CDR process. Despite oversight entities like the CADTH Pharmaceutical Advisory Committee Formulary Working Group, path dependent processes, linked to historical institutionalism, may lead to delays in policies catching up to evidence if a CDR is not triggered by manufacturers or if new evidence arises [110, 111]. Having taken an independent path, Quebec was not beholden to CADTH and the CDR recommendation to proceed with listing UPA-EC in their formulary. Furthermore, as a provincial body, the localized scope of INESSS may have facilitated the role of nurse prescribers in triggering the health technology assessment process for UPA-EC. Nevertheless, Health Canada appears to be revising their approach to the drug subsidization process, through an initiative aiming to align the recommendations of CADTH, INESSS and Health Canada [112]. Similarly, CADTH has been leading an Advisory Panel mandated with recommending a framework for developing a single, unifying nation-wide formulary [113].

### Strengths and limitations

We strengthened this policy analysis by employing multiple research methodologies, which provided deeper insight into the EC subsidization decision-making process in Canada. The documentary analysis included all ten provinces and drew data directly from the formularies, which are the gold standard source of information for this research question. This project allowed for knowledge sharing and collaboration between both parties involved in the semi-structured interview as key informants in turn asked questions and gained knowledge on the topic of EC.

Limitations include restricting the analysis to provincial formularies which may not capture the full experience of Canadian women obtaining EC. Provincial insurance plans often pertain to a limited fraction of the population, with many other Canadians obtaining their drug subsidizations from private insurance plans or paying out-of-pocket as they do not have access to any plans. Evaluating the policies in prominent private insurance companies could provide further understanding on the access experienced by Canadians. Although participating informants provided considerable insight, the limited sample size may lead to selection bias in the results. Pursuing snowball sampling techniques, stratifying selection by roles held in the decision-making process across several provinces and overall recruiting a larger sample size could increase the robustness of the qualitative results. The determination of this larger sample size would be guided by data saturation [60].

## Conclusions

This project has demonstrated that there is discrepancy between the evidence, which demonstrates that UPA-EC is the most effective oral EC method, and current subsidization policies in Canada. This gap may be explained by institutional structures which may interfere with actors’ ability to respond to the evolving EC literature. As Canada moves towards universal single-payer pharmacare, the question of whether to include a drug within this coverage will remain [114]. According to our findings, including UPA-EC in provincial formularies would be cost-effective and more equitable towards women of low income and higher body weights, providing more effective options to prevent unintended pregnancy. Involving a range of professionals such as nurses and pharmacists in delivering EC has been shown to facilitate implementation of policy, and to be reflective of the latest evidence on effectiveness of health professional roles. 

## Data Availability

The data analyzed during the current study are drawn from publicly available sources (provincial formularies, sexual clinic websites, university websites). The datasets generated and/or analysed during the current study are not publicly available due concerns surrounding confidentiality of participants who could be identified via the transcripts but are available from the corresponding author on reasonable request. Given the qualitative nature of this project, data availability would not be useful in allowing for reproducibility of the analysis and could infringe on the privacy of participants by sharing entire transcripts of the interviews.

## Abbreviations

BTC: Behind-the-counter
CADTH: Canadian Agency for Drugs and Technology in Health
CDR: Common Drug Review
Cu-IUD: Copper intrauterine device
EC: Emergency contraception
HCP: Healthcare professional
INESSS: Institut national d’excellence en santé et services sociaux
INSPQ: Institut national de santé publique du Québec
LNG-EC: Levonorgestrel emergency contraception
LNG-IUD: Levonorgestrel intrauterine device
OTC: Over-the-counter
UPA: Ulipristal acetate
UPA-EC: Ulipristal acetate emergency contraception
